# Supplementary material for: Development and validation of an interpretable longitudinal preeclampsia risk prediction using machine learning
Source: PLoS One. 2025 Jun 10;20(6):e0323873. doi: 10.1371/journal.pone.0323873 (PMC12151434; doi:10.1371/journal.pone.0323873)
Supplement: S1 File — Fig S1. Change in percentage of feature group contribution for model prediction throughout pregnancy. Fig S2. Interaction between individual features using Shapley values. Comparison between the xgboost model and the current standard of care as determined by ACOG guidelines. Table S1. Features used in the models. Table S2. Hyperparameter space for each model. Table S3. Best performing parameters for each model. Table S4. Characteristics of the external NuMoM2b dataset. Table S5. Equality of Opportunity results. Table S6. Model calibration. (DOCX) [file pone.0323873.s001.docx]

**Supporting Information**

**Development and validation of an interpretable longitudinal preeclampsia risk prediction using machine learning**

**AUTHORS:** Braden W Eberhard^1^, Raphael Y Cohen^1,2^, Nolan Wheeler^1^, Ricardo Kleinlein^1^, John Rigoni^1^, David W Bates^3,4^, Kathryn J Gray ^5*^, Vesela P Kovacheva^1*^

**Fig. S1.** Change in percentage of feature group contribution for model prediction throughout pregnancy.

**Fig. S2.** Interaction between individual features using Shapley values.

Comparison between the xgboost model and the current standard of care as determined by ACOG guidelines.

**Table S1.** Features used in the models.

**Table S2.** Hyperparameter space for each model.

**Table S3.** Best performing parameters for each model.

**Table S4.** Characteristics of the external NuMoM2b dataset.

**Table S5**. Equality of Opportunity results.

**Table S6.** Model calibration.

**Fig. S1.** Change in percentage of feature group contribution for preeclampsia model prediction throughout pregnancy.


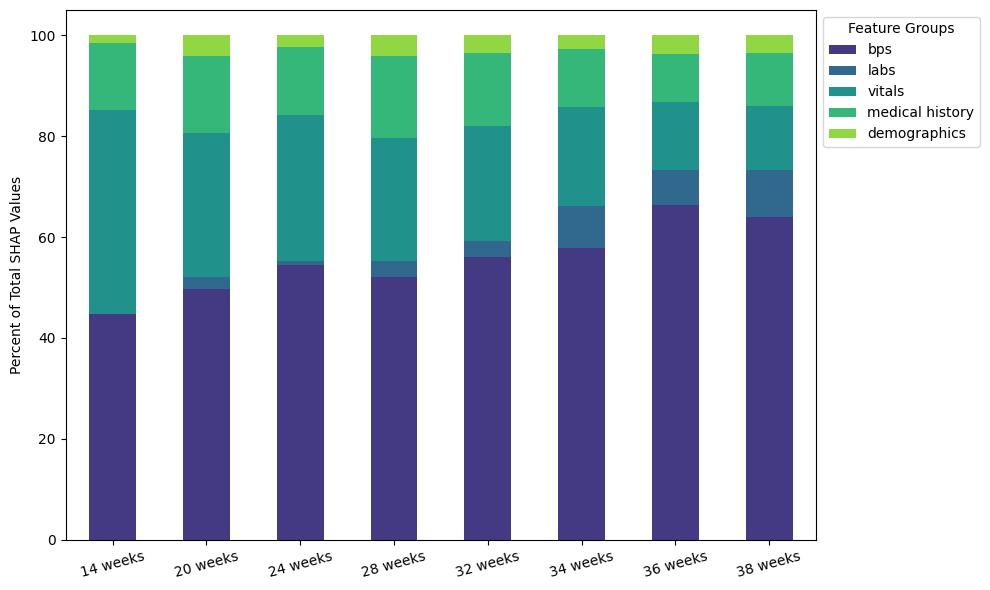


**Fig. S2. Interaction between individual features using Shapley values.** Every point is an individual delivery, and higher SHAP values show an increased effect toward a positive preeclampsia prediction. A variation on the y-axis at the same point on the x-axis reflects feature interaction. **A.** Relationship between the maternal age, nulliparity, and risk for preeclampsia. **B.** Relationship between maternal age, white race, and risk for preeclampsia. **C.** Relationship between mean systolic blood pressure, nulliparity, and risk for preeclampsia. **D.** Relationship between mean red blood cell count, maximum systolic blood pressure, and risk for preeclampsia.

| **S1 Table.** Features used in the models | | | | | | | | |
| --- | --- | --- | --- | --- | --- | --- | --- | --- |
| **Characteristic** | **Week 14** | **Week 20** | **Week 24** | **Week 28** | **Week 32** | **Week 34** | **Week 36** | **Week 38** |
| Autoimmune disease | all | all | all | all | all | all | all | all |
| baby_iugr | all | all | all | all | all | all | all | all |
| bmi_before_pregnancy__c3__lag_2 | all | all | all | all | all | all | all | all |
| bmi_before_pregnancy__length | all | all | all | all | all | all | all | all |
| bmi_before_pregnancy__maximum | nonlinear | nonlinear | nonlinear | nonlinear | nonlinear | nonlinear | nonlinear | nonlinear |
| bmi_before_pregnancy__mean | nonlinear | nonlinear | nonlinear | nonlinear | nonlinear | nonlinear | nonlinear | nonlinear |
| bmi_before_pregnancy__median | nonlinear | nonlinear | nonlinear | nonlinear | nonlinear | nonlinear | nonlinear | nonlinear |
| bmi_before_pregnancy__minimum | nonlinear | nonlinear | nonlinear | nonlinear | nonlinear | nonlinear | nonlinear | nonlinear |
| bmi_during_pregnancy__c3__lag_2 | all | all | all | nonlinear | nonlinear | nonlinear | nonlinear | nonlinear |
| bmi_during_pregnancy__length | nonlinear | nonlinear | nonlinear | nonlinear | nonlinear | nonlinear | nonlinear | nonlinear |
| bmi_during_pregnancy__maximum | nonlinear | nonlinear | nonlinear | nonlinear | nonlinear | nonlinear | nonlinear | nonlinear |
| bmi_during_pregnancy__mean | nonlinear | nonlinear | nonlinear | nonlinear | nonlinear | nonlinear | nonlinear | nonlinear |
| bmi_during_pregnancy__median | nonlinear | nonlinear | nonlinear | nonlinear | nonlinear | nonlinear | nonlinear | nonlinear |
| bmi_during_pregnancy__minimum | nonlinear | nonlinear | nonlinear | nonlinear | nonlinear | nonlinear | nonlinear | nonlinear |
| bmi_during_pregnancy_lin_coef | nonlinear | nonlinear | nonlinear | nonlinear | nonlinear | nonlinear | nonlinear | nonlinear |
| bmi_during_pregnancy_quad_coef | nonlinear | nonlinear | nonlinear | nonlinear | nonlinear | nonlinear | nonlinear | nonlinear |
| c_section_history | all | all | all | all | all | all | all | all |
| current_pregnancy_chypertension | all | all | all | all | all | all | all | all |
| current_pregnancy_gestational_diabetes | all | all | all | all | all | all | all | all |
| current_pregnancy_ghypertension | all | all | all | all | all | all | all | all |
| current_pregnancy_hyperemesis_gravidarum | all | all | all | all | all | all | all | all |
| current_pregnancy_iron_deficiency_anemia | all | all | all | all | all | all | all | all |
| current_pregnancy_pregnancy_related_fatigue | all | all | all | all | all | all | all | all |
| dbp__c3__lag_2 | nonlinear | nonlinear | nonlinear | nonlinear | nonlinear | nonlinear | nonlinear | nonlinear |
| dbp__length | none | none | nonlinear | nonlinear | none | nonlinear | nonlinear | none |
| dbp__maximum | nonlinear | nonlinear | nonlinear | nonlinear | nonlinear | nonlinear | nonlinear | nonlinear |
| dbp__mean | nonlinear | nonlinear | nonlinear | nonlinear | nonlinear | nonlinear | nonlinear | nonlinear |
| dbp__median | nonlinear | nonlinear | nonlinear | nonlinear | nonlinear | nonlinear | nonlinear | nonlinear |
| dbp__minimum | nonlinear | nonlinear | nonlinear | nonlinear | nonlinear | nonlinear | nonlinear | nonlinear |
| dbp_lin_coef | nonlinear | nonlinear | nonlinear | nonlinear | nonlinear | nonlinear | nonlinear | nonlinear |
| dbp_quad_coef | nonlinear | nonlinear | nonlinear | nonlinear | nonlinear | nonlinear | nonlinear | nonlinear |
| diabetes | all | all | all | all | all | all | all | all |
| drugs_or_alcohol | all | all | all | all | all | all | all | all |
| fam_history_of_icd_any_other_heart_disease | all | all | all | all | all | all | all | all |
| gravidity | all | all | all | all | all | all | all | all |
| high_risk | all | all | all | all | all | all | all | all |
| hr__c3__lag_2 | all | all | all | all | all | all | all | all |
| hr__maximum | nonlinear | nonlinear | nonlinear | nonlinear | nonlinear | nonlinear | nonlinear | nonlinear |
| hr__mean | nonlinear | nonlinear | nonlinear | nonlinear | nonlinear | nonlinear | nonlinear | nonlinear |
| hr__median | nonlinear | nonlinear | nonlinear | nonlinear | nonlinear | nonlinear | nonlinear | nonlinear |
| hr__minimum | nonlinear | nonlinear | nonlinear | nonlinear | nonlinear | nonlinear | nonlinear | nonlinear |
| hydramnios | all | all | all | all | all | all | all | all |
| ivf | all | all | all | all | all | all | all | all |
| kidney_disease | all | all | all | all | all | all | all | all |
| labs_calcium_serum__c3__lag_2 | all | all | all | all | all | all | all | all |
| labs_calcium_serum__maximum | none | nonlinear | none | none | nonlinear | nonlinear | nonlinear | nonlinear |
| labs_calcium_serum__mean | none | nonlinear | nonlinear | none | nonlinear | nonlinear | nonlinear | nonlinear |
| labs_calcium_serum__median | none | nonlinear | nonlinear | none | nonlinear | nonlinear | nonlinear | nonlinear |
| labs_creatinine_serum__minimum | none | none | none | none | none | none | none | nonlinear |
| labs_glucose_serum__mean | none | none | none | none | nonlinear | nonlinear | none | nonlinear |
| labs_glucose_serum__median | none | none | none | none | nonlinear | none | none | nonlinear |
| labs_glucose_serum__minimum | none | none | none | none | nonlinear | none | none | nonlinear |
| labs_hemoglobin__c3__lag_2 | nonlinear | nonlinear | nonlinear | nonlinear | nonlinear | nonlinear | nonlinear | nonlinear |
| labs_platelets__minimum | none | none | nonlinear | nonlinear | none | none | none | none |
| labs_rbc_count__c3__lag_2 | nonlinear | nonlinear | nonlinear | nonlinear | nonlinear | nonlinear | nonlinear | nonlinear |
| labs_rbc_count__maximum | nonlinear | nonlinear | nonlinear | nonlinear | nonlinear | nonlinear | nonlinear | nonlinear |
| labs_rbc_count__mean | nonlinear | nonlinear | nonlinear | nonlinear | nonlinear | nonlinear | nonlinear | nonlinear |
| labs_rbc_count__median | nonlinear | nonlinear | nonlinear | nonlinear | nonlinear | nonlinear | nonlinear | nonlinear |
| labs_rbc_count__minimum | nonlinear | nonlinear | nonlinear | nonlinear | nonlinear | nonlinear | nonlinear | nonlinear |
| labs_uric_acid__maximum | none | none | none | none | none | none | none | nonlinear |
| labs_uric_acid__mean | none | none | none | none | none | none | nonlinear | nonlinear |
| labs_uric_acid__median | none | none | none | none | none | nonlinear | nonlinear | nonlinear |
| labs_uric_acid__minimum | none | none | none | none | none | nonlinear | nonlinear | nonlinear |
| labs_urine_spot_protein_timeseries__length | none | none | none | none | none | none | all | all |
| labs_wbc_count__c3__lag_2 | none | none | none | none | none | none | all | all |
| labs_wbc_count__maximum | nonlinear | nonlinear | nonlinear | nonlinear | nonlinear | nonlinear | nonlinear | nonlinear |
| labs_wbc_count__mean | nonlinear | nonlinear | nonlinear | nonlinear | none | nonlinear | nonlinear | nonlinear |
| labs_wbc_count__median | nonlinear | nonlinear | nonlinear | nonlinear | none | nonlinear | nonlinear | nonlinear |
| labs_wbc_count__minimum | nonlinear | nonlinear | nonlinear | nonlinear | nonlinear | nonlinear | nonlinear | nonlinear |
| m_past_medical_history_heart_disease | all | all | all | all | all | all | all | all |
| maternal_age | nonlinear | nonlinear | nonlinear | nonlinear | nonlinear | nonlinear | nonlinear | nonlinear |
| minority | all | all | all | all | all | all | all | all |
| nulliparous | all | all | all | all | all | all | all | all |
| past_medical_history_chronic_hypertension | all | all | all | all | all | all | all | all |
| past_medical_history_migraine | all | all | all | all | all | all | all | all |
| past_medical_history_pregnancy_related_fatigue | all | all | all | all | all | all | all | all |
| past_medical_history_std | all | all | all | all | all | all | all | all |
| private_insurance | nonlinear | nonlinear | nonlinear | nonlinear | nonlinear | nonlinear | nonlinear | nonlinear |
| proteinuria | all | all | all | all | all | all | all | all |
| public_insurance | all | all | all | all | all | all | all | all |
| sbp_0-14_max | nonlinear | nonlinear | nonlinear | nonlinear | nonlinear | nonlinear | nonlinear | nonlinear |
| sbp_0-14_mean | nonlinear | nonlinear | nonlinear | nonlinear | nonlinear | nonlinear | nonlinear | nonlinear |
| sbp_0-14_min | nonlinear | nonlinear | nonlinear | nonlinear | nonlinear | nonlinear | nonlinear | nonlinear |
| sbp_0-14_vs_14-20_max_diff | none | nonlinear | nonlinear | nonlinear | nonlinear | nonlinear | nonlinear | nonlinear |
| sbp_0-14_vs_14-20_mean_diff | none | nonlinear | nonlinear | nonlinear | nonlinear | nonlinear | nonlinear | nonlinear |
| sbp_0-14_vs_14-20_min_diff | none | nonlinear | nonlinear | nonlinear | nonlinear | nonlinear | nonlinear | nonlinear |
| sbp_0-14_vs_20-24_max_diff | none | none | nonlinear | nonlinear | nonlinear | nonlinear | nonlinear | nonlinear |
| sbp_0-14_vs_20-24_mean_diff | none | none | nonlinear | nonlinear | nonlinear | nonlinear | nonlinear | nonlinear |
| sbp_0-14_vs_20-24_min_diff | none | none | nonlinear | nonlinear | nonlinear | nonlinear | nonlinear | nonlinear |
| sbp_0-14_vs_24-28_max_diff | none | none | none | nonlinear | nonlinear | nonlinear | nonlinear | nonlinear |
| sbp_0-14_vs_24-28_mean_diff | none | none | none | nonlinear | nonlinear | nonlinear | nonlinear | nonlinear |
| sbp_0-14_vs_24-28_min_diff | none | none | none | nonlinear | nonlinear | nonlinear | nonlinear | nonlinear |
| sbp_0-14_vs_28-32_max_diff | none | none | none | none | nonlinear | nonlinear | nonlinear | nonlinear |
| sbp_0-14_vs_28-32_mean_diff | none | none | none | none | nonlinear | nonlinear | nonlinear | nonlinear |
| sbp_0-14_vs_28-32_min_diff | none | none | none | none | nonlinear | nonlinear | nonlinear | nonlinear |
| sbp_0-14_vs_32-34_max_diff | none | none | none | none | none | nonlinear | nonlinear | nonlinear |
| sbp_0-14_vs_32-34_mean_diff | none | none | none | none | none | nonlinear | nonlinear | nonlinear |
| sbp_0-14_vs_32-34_min_diff | none | none | none | none | none | nonlinear | nonlinear | nonlinear |
| sbp_0-14_vs_34-36_max_diff | none | none | none | none | none | none | nonlinear | nonlinear |
| sbp_0-14_vs_34-36_mean_diff | none | none | none | none | none | none | nonlinear | nonlinear |
| sbp_0-14_vs_34-36_min_diff | none | none | none | none | none | none | nonlinear | nonlinear |
| sbp_0-14_vs_36-38_max_diff | none | none | none | none | none | none | none | nonlinear |
| sbp_0-14_vs_36-38_mean_diff | none | none | none | none | none | none | none | nonlinear |
| sbp_0-14_vs_36-38_min_diff | none | none | none | none | none | none | none | nonlinear |
| sbp_14-20_max | none | nonlinear | nonlinear | nonlinear | nonlinear | nonlinear | nonlinear | nonlinear |
| sbp_14-20_mean | none | nonlinear | nonlinear | nonlinear | nonlinear | nonlinear | nonlinear | nonlinear |
| sbp_14-20_min | none | nonlinear | nonlinear | nonlinear | nonlinear | nonlinear | nonlinear | nonlinear |
| sbp_14-20_vs_20-24_max_diff | none | none | nonlinear | nonlinear | nonlinear | nonlinear | nonlinear | nonlinear |
| sbp_14-20_vs_20-24_mean_diff | none | none | nonlinear | nonlinear | nonlinear | nonlinear | nonlinear | nonlinear |
| sbp_14-20_vs_20-24_min_diff | none | none | nonlinear | nonlinear | nonlinear | nonlinear | nonlinear | nonlinear |
| sbp_14-20_vs_24-28_max_diff | none | none | none | nonlinear | nonlinear | nonlinear | nonlinear | nonlinear |
| sbp_14-20_vs_24-28_mean_diff | none | none | none | nonlinear | nonlinear | nonlinear | nonlinear | nonlinear |
| sbp_14-20_vs_24-28_min_diff | none | none | none | nonlinear | nonlinear | nonlinear | nonlinear | nonlinear |
| sbp_14-20_vs_28-32_max_diff | none | none | none | none | nonlinear | nonlinear | nonlinear | nonlinear |
| sbp_14-20_vs_28-32_mean_diff | none | none | none | none | nonlinear | nonlinear | nonlinear | nonlinear |
| sbp_14-20_vs_28-32_min_diff | none | none | none | none | nonlinear | nonlinear | nonlinear | nonlinear |
| sbp_14-20_vs_32-34_max_diff | none | none | none | none | none | nonlinear | nonlinear | nonlinear |
| sbp_14-20_vs_32-34_mean_diff | none | none | none | none | none | nonlinear | nonlinear | nonlinear |
| sbp_14-20_vs_32-34_min_diff | none | none | none | none | none | nonlinear | nonlinear | nonlinear |
| sbp_14-20_vs_34-36_max_diff | none | none | none | none | none | none | nonlinear | nonlinear |
| sbp_14-20_vs_34-36_mean_diff | none | none | none | none | none | none | nonlinear | nonlinear |
| sbp_14-20_vs_34-36_min_diff | none | none | none | none | none | none | nonlinear | nonlinear |
| sbp_14-20_vs_36-38_max_diff | none | none | none | none | none | none | none | nonlinear |
| sbp_14-20_vs_36-38_mean_diff | none | none | none | none | none | none | none | nonlinear |
| sbp_14-20_vs_36-38_min_diff | none | none | none | none | none | none | none | nonlinear |
| sbp_20-24_max | none | none | nonlinear | nonlinear | nonlinear | nonlinear | nonlinear | nonlinear |
| sbp_20-24_mean | none | none | nonlinear | nonlinear | nonlinear | nonlinear | nonlinear | nonlinear |
| sbp_20-24_min | none | none | nonlinear | nonlinear | nonlinear | nonlinear | nonlinear | nonlinear |
| sbp_20-24_vs_24-28_max_diff | none | none | none | nonlinear | nonlinear | nonlinear | nonlinear | nonlinear |
| sbp_20-24_vs_24-28_mean_diff | none | none | none | nonlinear | nonlinear | nonlinear | nonlinear | nonlinear |
| sbp_20-24_vs_24-28_min_diff | none | none | none | nonlinear | nonlinear | nonlinear | nonlinear | nonlinear |
| sbp_20-24_vs_28-32_max_diff | none | none | none | none | nonlinear | nonlinear | nonlinear | nonlinear |
| sbp_20-24_vs_28-32_mean_diff | none | none | none | none | nonlinear | nonlinear | nonlinear | nonlinear |
| sbp_20-24_vs_28-32_min_diff | none | none | none | none | nonlinear | nonlinear | nonlinear | nonlinear |
| sbp_20-24_vs_32-34_max_diff | none | none | none | none | none | nonlinear | nonlinear | nonlinear |
| sbp_20-24_vs_32-34_mean_diff | none | none | none | none | none | nonlinear | nonlinear | nonlinear |
| sbp_20-24_vs_32-34_min_diff | none | none | none | none | none | nonlinear | nonlinear | nonlinear |
| sbp_20-24_vs_34-36_max_diff | none | none | none | none | none | none | nonlinear | nonlinear |
| sbp_20-24_vs_34-36_mean_diff | none | none | none | none | none | none | nonlinear | nonlinear |
| sbp_20-24_vs_34-36_min_diff | none | none | none | none | none | none | nonlinear | nonlinear |
| sbp_20-24_vs_36-38_max_diff | none | none | none | none | none | none | none | nonlinear |
| sbp_20-24_vs_36-38_mean_diff | none | none | none | none | none | none | none | nonlinear |
| sbp_20-24_vs_36-38_min_diff | none | none | none | none | none | none | none | nonlinear |
| sbp_24-28_max | none | none | none | nonlinear | nonlinear | nonlinear | nonlinear | nonlinear |
| sbp_24-28_mean | none | none | none | nonlinear | nonlinear | nonlinear | nonlinear | nonlinear |
| sbp_24-28_min | none | none | none | nonlinear | nonlinear | nonlinear | nonlinear | nonlinear |
| sbp_24-28_vs_28-32_max_diff | none | none | none | none | nonlinear | nonlinear | nonlinear | nonlinear |
| sbp_24-28_vs_28-32_mean_diff | none | none | none | none | nonlinear | nonlinear | nonlinear | nonlinear |
| sbp_24-28_vs_28-32_min_diff | none | none | none | none | nonlinear | nonlinear | nonlinear | nonlinear |
| sbp_24-28_vs_32-34_max_diff | none | none | none | none | none | nonlinear | nonlinear | nonlinear |
| sbp_24-28_vs_32-34_mean_diff | none | none | none | none | none | nonlinear | nonlinear | nonlinear |
| sbp_24-28_vs_32-34_min_diff | none | none | none | none | none | nonlinear | nonlinear | nonlinear |
| sbp_24-28_vs_34-36_max_diff | none | none | none | none | none | none | nonlinear | nonlinear |
| sbp_24-28_vs_34-36_mean_diff | none | none | none | none | none | none | nonlinear | nonlinear |
| sbp_24-28_vs_34-36_min_diff | none | none | none | none | none | none | nonlinear | nonlinear |
| sbp_24-28_vs_36-38_max_diff | none | none | none | none | none | none | none | nonlinear |
| sbp_24-28_vs_36-38_mean_diff | none | none | none | none | none | none | none | nonlinear |
| sbp_24-28_vs_36-38_min_diff | none | none | none | none | none | none | none | nonlinear |
| sbp_28-32_max | none | none | none | none | nonlinear | nonlinear | nonlinear | nonlinear |
| sbp_28-32_mean | none | none | none | none | nonlinear | nonlinear | nonlinear | nonlinear |
| sbp_28-32_min | none | none | none | none | nonlinear | nonlinear | nonlinear | nonlinear |
| sbp_28-32_vs_32-34_max_diff | none | none | none | none | none | nonlinear | nonlinear | nonlinear |
| sbp_28-32_vs_32-34_mean_diff | none | none | none | none | none | nonlinear | nonlinear | nonlinear |
| sbp_28-32_vs_32-34_min_diff | none | none | none | none | none | nonlinear | nonlinear | nonlinear |
| sbp_28-32_vs_34-36_max_diff | none | none | none | none | none | none | nonlinear | nonlinear |
| sbp_28-32_vs_34-36_mean_diff | none | none | none | none | none | none | nonlinear | nonlinear |
| sbp_28-32_vs_34-36_min_diff | none | none | none | none | none | none | nonlinear | nonlinear |
| sbp_28-32_vs_36-38_max_diff | none | none | none | none | none | none | none | nonlinear |
| sbp_28-32_vs_36-38_mean_diff | none | none | none | none | none | none | none | nonlinear |
| sbp_28-32_vs_36-38_min_diff | none | none | none | none | none | none | none | nonlinear |
| sbp_32-34_max | none | none | none | none | none | nonlinear | nonlinear | nonlinear |
| sbp_32-34_mean | none | none | none | none | none | nonlinear | nonlinear | nonlinear |
| sbp_32-34_min | none | none | none | none | none | nonlinear | nonlinear | nonlinear |
| sbp_32-34_vs_34-36_max_diff | none | none | none | none | none | none | nonlinear | nonlinear |
| sbp_32-34_vs_34-36_mean_diff | none | none | none | none | none | none | nonlinear | nonlinear |
| sbp_32-34_vs_34-36_min_diff | none | none | none | none | none | none | nonlinear | nonlinear |
| sbp_32-34_vs_36-38_max_diff | none | none | none | none | none | none | none | nonlinear |
| sbp_32-34_vs_36-38_mean_diff | none | none | none | none | none | none | none | nonlinear |
| sbp_32-34_vs_36-38_min_diff | none | none | none | none | none | none | none | nonlinear |
| sbp_34-36_max | none | none | none | none | none | none | nonlinear | nonlinear |
| sbp_34-36_mean | none | none | none | none | none | none | nonlinear | nonlinear |
| sbp_34-36_min | none | none | none | none | none | none | nonlinear | nonlinear |
| sbp_34-36_vs_36-38_max_diff | none | none | none | none | none | none | none | nonlinear |
| sbp_34-36_vs_36-38_mean_diff | none | none | none | none | none | none | none | nonlinear |
| sbp_34-36_vs_36-38_min_diff | none | none | none | none | none | none | none | nonlinear |
| sbp_36-38_max | none | none | none | none | none | none | none | nonlinear |
| sbp_36-38_mean | none | none | none | none | none | none | none | nonlinear |
| sbp_36-38_min | none | none | none | none | none | none | none | nonlinear |
| sbp__c3__lag_2 | nonlinear | nonlinear | nonlinear | nonlinear | nonlinear | nonlinear | nonlinear | nonlinear |
| sbp__length | none | none | nonlinear | nonlinear | none | nonlinear | nonlinear | none |
| sbp__maximum | nonlinear | nonlinear | nonlinear | nonlinear | nonlinear | nonlinear | nonlinear | nonlinear |
| sbp__mean | nonlinear | nonlinear | nonlinear | nonlinear | nonlinear | nonlinear | nonlinear | nonlinear |
| sbp__median | nonlinear | nonlinear | nonlinear | nonlinear | nonlinear | nonlinear | nonlinear | nonlinear |
| sbp__minimum | nonlinear | nonlinear | nonlinear | nonlinear | nonlinear | nonlinear | nonlinear | nonlinear |
| sbp_lin_coef | nonlinear | nonlinear | nonlinear | nonlinear | nonlinear | nonlinear | nonlinear | nonlinear |
| sbp_quad_coef | nonlinear | nonlinear | nonlinear | nonlinear | nonlinear | nonlinear | nonlinear | nonlinear |
| weight_before_pregnancy__c3__lag_2 | all | all | all | all | all | all | all | all |
| weight_before_pregnancy__length | nonlinear | nonlinear | nonlinear | nonlinear | nonlinear | nonlinear | nonlinear | nonlinear |
| weight_before_pregnancy__maximum | nonlinear | nonlinear | nonlinear | nonlinear | nonlinear | nonlinear | nonlinear | nonlinear |
| weight_before_pregnancy__mean | nonlinear | nonlinear | nonlinear | nonlinear | nonlinear | nonlinear | nonlinear | nonlinear |
| weight_before_pregnancy__median | nonlinear | nonlinear | nonlinear | nonlinear | nonlinear | nonlinear | nonlinear | nonlinear |
| weight_before_pregnancy__minimum | nonlinear | nonlinear | nonlinear | nonlinear | nonlinear | nonlinear | nonlinear | nonlinear |
| weight_during_pregnancy__c3__lag_2 | none | all | all | nonlinear | nonlinear | nonlinear | nonlinear | nonlinear |
| weight_during_pregnancy__length | nonlinear | nonlinear | nonlinear | nonlinear | nonlinear | nonlinear | nonlinear | nonlinear |
| weight_during_pregnancy__maximum | nonlinear | nonlinear | nonlinear | nonlinear | nonlinear | nonlinear | nonlinear | nonlinear |
| weight_during_pregnancy__mean | nonlinear | nonlinear | nonlinear | nonlinear | nonlinear | nonlinear | nonlinear | nonlinear |
| weight_during_pregnancy__median | nonlinear | nonlinear | nonlinear | nonlinear | nonlinear | nonlinear | nonlinear | nonlinear |
| weight_during_pregnancy__minimum | nonlinear | nonlinear | nonlinear | nonlinear | nonlinear | nonlinear | nonlinear | nonlinear |
| white | nonlinear | nonlinear | nonlinear | nonlinear | nonlinear | nonlinear | nonlinear | nonlinear |
| Linear models: logistic regression, elastic net  Nonlinear models: naïve Bayes, random forest, xgboost, neural network | | | | | | | | |

| **S2 Table.** Hyperparameter space for each model. | |
| --- | --- |
| **Model** | **Parameter_Space** |
| Random forest | random_state: 0  max_depth: int[3, 10]  n_estimators: int[50, 200]  min_samples_split: int [2, 20] |
| xgboost | seed: 0  use_label_encoder: False  eval_metric: logloss  max_depth: int[1, 4]  learning_rate: loguniform[0.001, 0.1] |
| Logistic regression | random_state: 0  penalty: l2  solver: lbfgs  max_iter: 100  C: loguniform[0.001,10] |
| Elastic net | random_state: 0  max_iter: 100  alpha: loguniform[0.001, 0.1] |
| naive_bayes | var_smoothing: loguniform[1e-9, 1e-7] |
| Multi-layer perceptron | random_state: 0  activation: categorical[ 'identity', 'logistic', 'tanh', 'relu']  solver: categorical[ 'lbfgs', 'sgd', 'adam']  alpha: loguniform[1e-5, 1e-3]  learning_rate: categorical['constant', 'invscaling', 'adaptive']  max_iter: 20  tol: 0.0001 |

| **S3 Table**. Best performing parameters for each preeclampsia prediction model. | | | | | | |
| --- | --- | --- | --- | --- | --- | --- |
| **Weeks** | **Elastic net** | **Logistic regression** | **Multi-layer perceptron** | **naive_bayes** | **Random forest** | **xgboost** |
| 14 | alpha: 0.00192 | logistic_C: 0.405 | mlp_activation: logistic, mlp_alpha: 0.000259, mlp_hidden_layer_0: 111, mlp_hidden_layer_1: 190, mlp_hidden_layer_2: 197, mlp_learning_rate: constant, mlp_n_layers: 3, mlp_solver: adam | naive_bayes_var_smoothing: 7.69e-09 | max_depth: 8, min_samples_split: 5, n_estimators: 169 | lr: 0.085, max_depth: 1, n_estimators: 75 |
| 20 | alpha: 0.00108 | logistic_C: 0.33 | mlp_activation: logistic, mlp_alpha: 3.34e-05, mlp_hidden_layer_0: 72, mlp_hidden_layer_1: 94, mlp_hidden_layer_2: 186, mlp_learning_rate: constant, mlp_n_layers: 3, mlp_solver: adam | naive_bayes_var_smoothing: 9.04e-08 | max_depth: 8, min_samples_split: 3, n_estimators: 89 | lr: 0.0777, max_depth: 4, n_estimators: 75 |
| 24 | alpha: 0.00108 | logistic_C: 0.351 | mlp_activation: logistic, mlp_alpha: 0.000769, mlp_hidden_layer_0: 85, mlp_hidden_layer_1: 129, mlp_learning_rate: invscaling, mlp_n_layers: 2, mlp_solver: adam | naive_bayes_var_smoothing: 8.06e-08 | max_depth: 9, min_samples_split: 20, n_estimators: 200 | lr: 0.0493, max_depth: 3, n_estimators: 50 |
| 28 | alpha: 0.00415 | logistic_C: 0.1 | mlp_activation: identity, mlp_alpha: 0.000148, mlp_hidden_layer_0: 82, mlp_learning_rate: constant, mlp_n_layers: 1, mlp_solver: adam | naive_bayes_var_smoothing: 2.2e-09 | max_depth: 8, min_samples_split: 5, n_estimators: 164 | lr: 0.0629, max_depth: 4, n_estimators: 100 |
| 32 | alpha: 0.00208 | logistic_C: 0.381 | mlp_activation: identity, mlp_alpha: 0.000413, mlp_hidden_layer_0: 65, mlp_hidden_layer_1: 122, mlp_learning_rate: invscaling, mlp_n_layers: 2, mlp_solver: adam | naive_bayes_var_smoothing: 8.7e-08 | max_depth: 8, min_samples_split: 10, n_estimators: 75 | lr: 0.0744, max_depth: 2, n_estimators: 200 |
| 34 | alpha: 0.00144 | logistic_C: 0.256 | mlp_activation: identity, mlp_alpha: 1.61e-05, mlp_hidden_layer_0: 185, mlp_hidden_layer_1: 86, mlp_hidden_layer_2: 50, mlp_learning_rate: invscaling, mlp_n_layers: 3, mlp_solver: adam | naive_bayes_var_smoothing: 1.31e-09 | max_depth: 8, min_samples_split: 2, n_estimators: 153 | lr: 0.0827, max_depth: 2, n_estimators: 200 |
| 36 | alpha: 0.00171 | logistic_C: 0.17 | mlp_activation: tanh, mlp_alpha: 0.000216, mlp_hidden_layer_0: 197, mlp_hidden_layer_1: 78, mlp_learning_rate: constant, mlp_n_layers: 2, mlp_solver: sgd | naive_bayes_var_smoothing: 1.5e-09 | max_depth: 8, min_samples_split: 3, n_estimators: 140 | lr: 0.0509, max_depth: 4, n_estimators: 75 |
| 38 | alpha: 0.00141 | logistic_C: 0.185 | mlp_activation: relu, mlp_alpha: 0.000137, mlp_hidden_layer_0: 137, mlp_hidden_layer_1: 120, mlp_learning_rate: constant, mlp_n_layers: 2, mlp_solver: sgd | naive_bayes_var_smoothing: 3.1e-08 | max_depth: 8, min_samples_split: 3, n_estimators: 151 | lr: 0.0504, max_depth: 3, n_estimators: 200 |

| **S4 Table.** Characteristics of the external NuMoM2b dataset. | | | | |
| --- | --- | --- | --- | --- |
| **Characteristic** | **No preeclampsia n=8009** | **Preeclampsia**  **n=536** | **Total**  **n=8545** | **P value** |
| Maternal age at delivery, y | 27.38 ± 5.79 | 28.05 ± 5.63 | 28.01 ± 5.64 | 0.008 |
| Self-reported race, White | 310 (57.8%) | 5405 (67.5%) | 5715 (66.9%) | <0.001 |
| Self-reported race, Black | 123 (22.9%) | 1026 (12.8%) | 1149 (13.4%) | <0.001 |
| Self-reported race, other | 87 (16.2%) | 1235 (15.4%) | 1322 (15.5%) | 0.66 |
| Self-reported ethnicity, Hispanic | 90 (16.8%) | 1351 (16.9%) | 1441 (16.9%) | 1.0 |
| Self-reported ethnicity, non-Hispanic | 446 (83.2%) | 6658 (83.1%) | 7104 (83.1%) | 1.0 |
| Gravidity | 1.40 ± 0.71 | 1.33 ± 0.66 | 1.33 ± 0.66 | 0.02 |
| In vitro fertilization | 22 (4.1%) | 316 (3.9%) | 338 (4.0%) | 0.95 |
| Preterm delivery | 124 (23.1%) | 357 (4.5%) | 481 (5.6%) | <0.001 |
| Gestational age at delivery, weeks | 260.45 ± 22.87 | 274.72 ± 16.65 | 273.83 ± 17.45 | <0.001 |
| BMI at delivery, kg/m^2^ | 34.40 ± 7.93 | 30.45 ± 6.41 | 30.70 ± 6.59 | <0.001 |
| Weight at delivery, kg | 92.18 ± 22.72 | 82.45 ± 18.62 | 83.06 ± 19.05 | <0.001 |
| Maximal SBP during pregnancy, mmHg | 156.76 ± 16.84 | 121.39 ± 16.42 | 123.62 ± 18.55 | <0.001 |
| Maximal DBP during pregnancy, mmHg | 95.66 ± 10.77 | 74.78 ± 11.07 | 76.09 ± 12.16 | <0.001 |
| Family history of preeclampsia | 64 (11.9%) | 799 (10.0%) | 863 (10.1%) | 0.17 |
| Past history of chronic hypertension | 74 (13.8%) | 938 (11.7%) | 1012 (11.8%) | 0.17 |
| Antihypertensive medications throughout pregnancy and 6 weeks postpartum | 160 (29.9%) | 161 (2.0%) | 321 (3.8%) | <0.001 |
| Gestational diabetes | 3 (0.6%) | 63 (0.8%) | 66 (0.8%) | 0.74 |
| Proteinuria | 392 (73.1%) | 36 (0.4%) | 428 (5.0%) | <0.001 |
| Maximal uric acid during pregnancy | 5.65 ± 1.45 | 5.10 ± 1.31 | 5.31 ± 1.39 | <0.001 |
| SGA or IUGR | 27 (5.0%) | 194 (2.4%) | 221 (2.6%) | <0.001 |

Mean ± standard deviation for continuous variables; n (%) for categorical variables; p-values for continuous variables based on Kruskal-Wallis rank sum test; for categorical variables based on Fisher’s exact or Chi-squared test.

Abbreviations: SBP, systolic blood pressure; DBP, diastolic blood pressure, BMI, body mass index, SGA, small for gestational age, IUGR, intrauterine growth restriction.

| **S5 Table.** Equality of Opportunity results. | | | | |
| --- | --- | --- | --- | --- |
| **Gestational week** | **Group 1** | **Group 2** | **Equal opportunity difference** | **P value** |
| 14 | black | white | 0.159 | 0.521 |
| 14 | black | hispanic | 0.096 | 0.506 |
| 14 | black | public_insurance | 0.065 | 0.502 |
| 14 | black | private_insurance | 0.166 | 0.498 |
| 14 | black | drugs_or_alcohol | 0.127 | 0.503 |
| 14 | white | black | -0.159 | 0.484 |
| 14 | white | hispanic | -0.063 | 0.507 |
| 14 | white | public_insurance | -0.094 | 0.533 |
| 14 | white | private_insurance | 0.006 | 0.504 |
| 14 | white | drugs_or_alcohol | -0.032 | 0.488 |
| 14 | hispanic | black | -0.096 | 0.511 |
| 14 | hispanic | white | 0.063 | 0.5 |
| 14 | hispanic | public_insurance | -0.031 | 0.491 |
| 14 | hispanic | private_insurance | 0.069 | 0.498 |
| 14 | hispanic | drugs_or_alcohol | 0.031 | 0.499 |
| 14 | public_insurance | black | -0.065 | 0.515 |
| 14 | public_insurance | white | 0.094 | 0.505 |
| 14 | public_insurance | hispanic | 0.031 | 0.495 |
| 14 | public_insurance | private_insurance | 0.101 | 0.5 |
| 14 | public_insurance | drugs_or_alcohol | 0.062 | 0.509 |
| 14 | private_insurance | black | -0.166 | 0.472 |
| 14 | private_insurance | white | -0.006 | 0.52 |
| 14 | private_insurance | hispanic | -0.069 | 0.536 |
| 14 | private_insurance | public_insurance | -0.101 | 0.491 |
| 14 | private_insurance | drugs_or_alcohol | -0.039 | 0.5 |
| 14 | drugs_or_alcohol | black | -0.127 | 0.487 |
| 14 | drugs_or_alcohol | white | 0.032 | 0.486 |
| 14 | drugs_or_alcohol | hispanic | -0.031 | 0.51 |
| 14 | drugs_or_alcohol | public_insurance | -0.062 | 0.49 |
| 14 | drugs_or_alcohol | private_insurance | 0.039 | 0.471 |
| 20 | black | white | 0.214 | 0.518 |
| 20 | black | hispanic | 0.102 | 0.508 |
| 20 | black | public_insurance | 0.073 | 0.482 |
| 20 | black | private_insurance | 0.208 | 0.53 |
| 20 | black | drugs_or_alcohol | 0.174 | 0.494 |
| 20 | white | black | -0.214 | 0.481 |
| 20 | white | hispanic | -0.112 | 0.502 |
| 20 | white | public_insurance | -0.14 | 0.494 |
| 20 | white | private_insurance | -0.006 | 0.529 |
| 20 | white | drugs_or_alcohol | -0.04 | 0.487 |
| 20 | hispanic | black | -0.102 | 0.482 |
| 20 | hispanic | white | 0.112 | 0.499 |
| 20 | hispanic | public_insurance | -0.028 | 0.513 |
| 20 | hispanic | private_insurance | 0.106 | 0.494 |
| 20 | hispanic | drugs_or_alcohol | 0.072 | 0.502 |
| 20 | public_insurance | black | -0.073 | 0.469 |
| 20 | public_insurance | white | 0.14 | 0.476 |
| 20 | public_insurance | hispanic | 0.028 | 0.493 |
| 20 | public_insurance | private_insurance | 0.135 | 0.486 |
| 20 | public_insurance | drugs_or_alcohol | 0.101 | 0.504 |
| 20 | private_insurance | black | -0.208 | 0.478 |
| 20 | private_insurance | white | 0.006 | 0.516 |
| 20 | private_insurance | hispanic | -0.106 | 0.472 |
| 20 | private_insurance | public_insurance | -0.135 | 0.476 |
| 20 | private_insurance | drugs_or_alcohol | -0.034 | 0.491 |
| 20 | drugs_or_alcohol | black | -0.174 | 0.514 |
| 20 | drugs_or_alcohol | white | 0.04 | 0.528 |
| 20 | drugs_or_alcohol | hispanic | -0.072 | 0.497 |
| 20 | drugs_or_alcohol | public_insurance | -0.101 | 0.508 |
| 20 | drugs_or_alcohol | private_insurance | 0.034 | 0.5 |
| 24 | black | white | 0.156 | 0.473 |
| 24 | black | hispanic | 0.088 | 0.519 |
| 24 | black | public_insurance | 0.066 | 0.495 |
| 24 | black | private_insurance | 0.164 | 0.495 |
| 24 | black | drugs_or_alcohol | 0.13 | 0.5 |
| 24 | white | black | -0.156 | 0.48 |
| 24 | white | hispanic | -0.068 | 0.499 |
| 24 | white | public_insurance | -0.09 | 0.516 |
| 24 | white | private_insurance | 0.009 | 0.493 |
| 24 | white | drugs_or_alcohol | -0.026 | 0.501 |
| 24 | hispanic | black | -0.088 | 0.503 |
| 24 | hispanic | white | 0.068 | 0.461 |
| 24 | hispanic | public_insurance | -0.022 | 0.51 |
| 24 | hispanic | private_insurance | 0.077 | 0.5 |
| 24 | hispanic | drugs_or_alcohol | 0.042 | 0.501 |
| 24 | public_insurance | black | -0.066 | 0.496 |
| 24 | public_insurance | white | 0.09 | 0.498 |
| 24 | public_insurance | hispanic | 0.022 | 0.472 |
| 24 | public_insurance | private_insurance | 0.099 | 0.499 |
| 24 | public_insurance | drugs_or_alcohol | 0.064 | 0.498 |
| 24 | private_insurance | black | -0.164 | 0.518 |
| 24 | private_insurance | white | -0.009 | 0.474 |
| 24 | private_insurance | hispanic | -0.077 | 0.489 |
| 24 | private_insurance | public_insurance | -0.099 | 0.496 |
| 24 | private_insurance | drugs_or_alcohol | -0.035 | 0.491 |
| 24 | drugs_or_alcohol | black | -0.13 | 0.513 |
| 24 | drugs_or_alcohol | white | 0.026 | 0.523 |
| 24 | drugs_or_alcohol | hispanic | -0.042 | 0.483 |
| 24 | drugs_or_alcohol | public_insurance | -0.064 | 0.526 |
| 24 | drugs_or_alcohol | private_insurance | 0.035 | 0.492 |
| 28 | black | white | 0.189 | 0.487 |
| 28 | black | hispanic | 0.11 | 0.497 |
| 28 | black | public_insurance | 0.06 | 0.524 |
| 28 | black | private_insurance | 0.198 | 0.491 |
| 28 | black | drugs_or_alcohol | 0.151 | 0.486 |
| 28 | white | black | -0.189 | 0.525 |
| 28 | white | hispanic | -0.079 | 0.51 |
| 28 | white | public_insurance | -0.129 | 0.482 |
| 28 | white | private_insurance | 0.009 | 0.497 |
| 28 | white | drugs_or_alcohol | -0.038 | 0.484 |
| 28 | hispanic | black | -0.11 | 0.505 |
| 28 | hispanic | white | 0.079 | 0.5 |
| 28 | hispanic | public_insurance | -0.05 | 0.511 |
| 28 | hispanic | private_insurance | 0.088 | 0.507 |
| 28 | hispanic | drugs_or_alcohol | 0.041 | 0.5 |
| 28 | public_insurance | black | -0.06 | 0.501 |
| 28 | public_insurance | white | 0.129 | 0.506 |
| 28 | public_insurance | hispanic | 0.05 | 0.501 |
| 28 | public_insurance | private_insurance | 0.138 | 0.505 |
| 28 | public_insurance | drugs_or_alcohol | 0.091 | 0.503 |
| 28 | private_insurance | black | -0.198 | 0.503 |
| 28 | private_insurance | white | -0.009 | 0.523 |
| 28 | private_insurance | hispanic | -0.088 | 0.509 |
| 28 | private_insurance | public_insurance | -0.138 | 0.5 |
| 28 | private_insurance | drugs_or_alcohol | -0.047 | 0.518 |
| 28 | drugs_or_alcohol | black | -0.151 | 0.504 |
| 28 | drugs_or_alcohol | white | 0.038 | 0.509 |
| 28 | drugs_or_alcohol | hispanic | -0.041 | 0.494 |
| 28 | drugs_or_alcohol | public_insurance | -0.091 | 0.525 |
| 28 | drugs_or_alcohol | private_insurance | 0.047 | 0.516 |
| 32 | black | white | 0.212 | 0.5 |
| 32 | black | hispanic | 0.114 | 0.51 |
| 32 | black | public_insurance | 0.077 | 0.48 |
| 32 | black | private_insurance | 0.207 | 0.514 |
| 32 | black | drugs_or_alcohol | 0.169 | 0.511 |
| 32 | white | black | -0.212 | 0.511 |
| 32 | white | hispanic | -0.098 | 0.501 |
| 32 | white | public_insurance | -0.135 | 0.502 |
| 32 | white | private_insurance | -0.006 | 0.537 |
| 32 | white | drugs_or_alcohol | -0.043 | 0.473 |
| 32 | hispanic | black | -0.114 | 0.532 |
| 32 | hispanic | white | 0.098 | 0.49 |
| 32 | hispanic | public_insurance | -0.037 | 0.49 |
| 32 | hispanic | private_insurance | 0.092 | 0.512 |
| 32 | hispanic | drugs_or_alcohol | 0.055 | 0.487 |
| 32 | public_insurance | black | -0.077 | 0.499 |
| 32 | public_insurance | white | 0.135 | 0.51 |
| 32 | public_insurance | hispanic | 0.037 | 0.485 |
| 32 | public_insurance | private_insurance | 0.13 | 0.499 |
| 32 | public_insurance | drugs_or_alcohol | 0.092 | 0.485 |
| 32 | private_insurance | black | -0.207 | 0.49 |
| 32 | private_insurance | white | 0.006 | 0.499 |
| 32 | private_insurance | hispanic | -0.092 | 0.489 |
| 32 | private_insurance | public_insurance | -0.13 | 0.503 |
| 32 | private_insurance | drugs_or_alcohol | -0.037 | 0.49 |
| 32 | drugs_or_alcohol | black | -0.169 | 0.492 |
| 32 | drugs_or_alcohol | white | 0.043 | 0.491 |
| 32 | drugs_or_alcohol | hispanic | -0.055 | 0.5 |
| 32 | drugs_or_alcohol | public_insurance | -0.092 | 0.524 |
| 32 | drugs_or_alcohol | private_insurance | 0.037 | 0.507 |
| 34 | black | white | 0.174 | 0.511 |
| 34 | black | hispanic | 0.097 | 0.52 |
| 34 | black | public_insurance | 0.067 | 0.511 |
| 34 | black | private_insurance | 0.17 | 0.518 |
| 34 | black | drugs_or_alcohol | 0.131 | 0.514 |
| 34 | white | black | -0.174 | 0.51 |
| 34 | white | hispanic | -0.077 | 0.517 |
| 34 | white | public_insurance | -0.107 | 0.505 |
| 34 | white | private_insurance | -0.004 | 0.564 |
| 34 | white | drugs_or_alcohol | -0.043 | 0.542 |
| 34 | hispanic | black | -0.097 | 0.513 |
| 34 | hispanic | white | 0.077 | 0.505 |
| 34 | hispanic | public_insurance | -0.03 | 0.496 |
| 34 | hispanic | private_insurance | 0.073 | 0.492 |
| 34 | hispanic | drugs_or_alcohol | 0.034 | 0.507 |
| 34 | public_insurance | black | -0.067 | 0.5 |
| 34 | public_insurance | white | 0.107 | 0.472 |
| 34 | public_insurance | hispanic | 0.03 | 0.52 |
| 34 | public_insurance | private_insurance | 0.103 | 0.49 |
| 34 | public_insurance | drugs_or_alcohol | 0.064 | 0.501 |
| 34 | private_insurance | black | -0.17 | 0.508 |
| 34 | private_insurance | white | 0.004 | 0.587 |
| 34 | private_insurance | hispanic | -0.073 | 0.516 |
| 34 | private_insurance | public_insurance | -0.103 | 0.497 |
| 34 | private_insurance | drugs_or_alcohol | -0.039 | 0.504 |
| 34 | drugs_or_alcohol | black | -0.131 | 0.493 |
| 34 | drugs_or_alcohol | white | 0.043 | 0.465 |
| 34 | drugs_or_alcohol | hispanic | -0.034 | 0.483 |
| 34 | drugs_or_alcohol | public_insurance | -0.064 | 0.529 |
| 34 | drugs_or_alcohol | private_insurance | 0.039 | 0.518 |
| 36 | black | white | 0.148 | 0.51 |
| 36 | black | hispanic | 0.113 | 0.457 |
| 36 | black | public_insurance | 0.092 | 0.508 |
| 36 | black | private_insurance | 0.148 | 0.508 |
| 36 | black | drugs_or_alcohol | 0.114 | 0.517 |
| 36 | white | black | -0.148 | 0.495 |
| 36 | white | hispanic | -0.036 | 0.512 |
| 36 | white | public_insurance | -0.057 | 0.505 |
| 36 | white | private_insurance | -0.0 | 0.96 |
| 36 | white | drugs_or_alcohol | -0.034 | 0.511 |
| 36 | hispanic | black | -0.113 | 0.519 |
| 36 | hispanic | white | 0.036 | 0.483 |
| 36 | hispanic | public_insurance | -0.021 | 0.505 |
| 36 | hispanic | private_insurance | 0.036 | 0.52 |
| 36 | hispanic | drugs_or_alcohol | 0.002 | 0.878 |
| 36 | public_insurance | black | -0.092 | 0.529 |
| 36 | public_insurance | white | 0.057 | 0.5 |
| 36 | public_insurance | hispanic | 0.021 | 0.527 |
| 36 | public_insurance | private_insurance | 0.056 | 0.504 |
| 36 | public_insurance | drugs_or_alcohol | 0.022 | 0.497 |
| 36 | private_insurance | black | -0.148 | 0.519 |
| 36 | private_insurance | white | 0.0 | 0.954 |
| 36 | private_insurance | hispanic | -0.036 | 0.507 |
| 36 | private_insurance | public_insurance | -0.056 | 0.507 |
| 36 | private_insurance | drugs_or_alcohol | -0.034 | 0.5 |
| 36 | drugs_or_alcohol | black | -0.114 | 0.477 |
| 36 | drugs_or_alcohol | white | 0.034 | 0.497 |
| 36 | drugs_or_alcohol | hispanic | -0.002 | 0.876 |
| 36 | drugs_or_alcohol | public_insurance | -0.022 | 0.494 |
| 36 | drugs_or_alcohol | private_insurance | 0.034 | 0.515 |
| 38 | black | white | 0.21 | 0.494 |
| 38 | black | hispanic | 0.116 | 0.506 |
| 38 | black | public_insurance | 0.099 | 0.51 |
| 38 | black | private_insurance | 0.197 | 0.538 |
| 38 | black | drugs_or_alcohol | 0.179 | 0.51 |
| 38 | white | black | -0.21 | 0.51 |
| 38 | white | hispanic | -0.094 | 0.49 |
| 38 | white | public_insurance | -0.111 | 0.498 |
| 38 | white | private_insurance | -0.012 | 0.499 |
| 38 | white | drugs_or_alcohol | -0.03 | 0.486 |
| 38 | hispanic | black | -0.116 | 0.517 |
| 38 | hispanic | white | 0.094 | 0.502 |
| 38 | hispanic | public_insurance | -0.017 | 0.496 |
| 38 | hispanic | private_insurance | 0.082 | 0.5 |
| 38 | hispanic | drugs_or_alcohol | 0.064 | 0.512 |
| 38 | public_insurance | black | -0.099 | 0.502 |
| 38 | public_insurance | white | 0.111 | 0.501 |
| 38 | public_insurance | hispanic | 0.017 | 0.507 |
| 38 | public_insurance | private_insurance | 0.098 | 0.509 |
| 38 | public_insurance | drugs_or_alcohol | 0.08 | 0.499 |
| 38 | private_insurance | black | -0.197 | 0.51 |
| 38 | private_insurance | white | 0.012 | 0.509 |
| 38 | private_insurance | hispanic | -0.082 | 0.502 |
| 38 | private_insurance | public_insurance | -0.098 | 0.477 |
| 38 | private_insurance | drugs_or_alcohol | -0.018 | 0.482 |
| 38 | drugs_or_alcohol | black | -0.179 | 0.503 |
| 38 | drugs_or_alcohol | white | 0.03 | 0.488 |
| 38 | drugs_or_alcohol | hispanic | -0.064 | 0.504 |
| 38 | drugs_or_alcohol | public_insurance | -0.08 | 0.492 |
| 38 | drugs_or_alcohol | private_insurance | 0.018 | 0.501 |

| **S6 Table.** Preeclampsia model calibration | | | | | | |
| --- | --- | --- | --- | --- | --- | --- |
| **Gestational weeks** | **Threshold** | **Alarm Rate** | **PPV** | **NPV** | **Sensitivity** | **Specificity** |
| 14 weeks | **0.3** | 0.78 | 0.074 | 0.985 | 0.945 | 0.23 |
|  | **0.4** | 0.508 | 0.097 | 0.976 | 0.808 | 0.512 |
|  | **0.5** | 0.264 | 0.147 | 0.969 | 0.633 | 0.76 |
|  | **0.6** | 0.129 | 0.219 | 0.962 | 0.463 | 0.893 |
|  | **0.7** | 0.06 | 0.313 | 0.955 | 0.308 | 0.956 |
| 20 weeks | **0.3** | 0.624 | 0.087 | 0.985 | 0.904 | 0.394 |
|  | **0.4** | 0.465 | 0.106 | 0.98 | 0.823 | 0.558 |
|  | **0.5** | 0.283 | 0.146 | 0.974 | 0.688 | 0.743 |
|  | **0.6** | 0.159 | 0.197 | 0.966 | 0.522 | 0.864 |
|  | **0.7** | 0.087 | 0.264 | 0.959 | 0.383 | 0.932 |
| 24 weeks | **0.3** | 0.72 | 0.081 | 0.988 | 0.946 | 0.295 |
|  | **0.4** | 0.512 | 0.103 | 0.982 | 0.855 | 0.511 |
|  | **0.5** | 0.277 | 0.147 | 0.971 | 0.661 | 0.748 |
|  | **0.6** | 0.149 | 0.195 | 0.962 | 0.472 | 0.872 |
|  | **0.7** | 0.068 | 0.282 | 0.954 | 0.312 | 0.948 |
| 28 weeks | **0.3** | 0.592 | 0.091 | 0.988 | 0.918 | 0.428 |
|  | **0.4** | 0.453 | 0.109 | 0.983 | 0.838 | 0.571 |
|  | **0.5** | 0.278 | 0.146 | 0.975 | 0.69 | 0.747 |
|  | **0.6** | 0.161 | 0.196 | 0.968 | 0.538 | 0.862 |
|  | **0.7** | 0.089 | 0.263 | 0.961 | 0.398 | 0.93 |
| 32 weeks | **0.3** | 0.599 | 0.089 | 0.988 | 0.917 | 0.42 |
|  | **0.4** | 0.441 | 0.108 | 0.982 | 0.824 | 0.582 |
|  | **0.5** | 0.277 | 0.144 | 0.975 | 0.687 | 0.749 |
|  | **0.6** | 0.164 | 0.194 | 0.969 | 0.552 | 0.859 |
|  | **0.7** | 0.091 | 0.252 | 0.962 | 0.398 | 0.927 |
| 34 weeks | **0.3** | 0.574 | 0.08 | 0.988 | 0.901 | 0.443 |
|  | **0.4** | 0.42 | 0.099 | 0.984 | 0.821 | 0.601 |
|  | **0.5** | 0.274 | 0.131 | 0.979 | 0.705 | 0.749 |
|  | **0.6** | 0.168 | 0.173 | 0.974 | 0.57 | 0.853 |
|  | **0.7** | 0.09 | 0.227 | 0.966 | 0.401 | 0.927 |
| 36 weeks | **0.3** | 0.567 | 0.073 | 0.989 | 0.898 | 0.449 |
|  | **0.4** | 0.426 | 0.089 | 0.985 | 0.816 | 0.593 |
|  | **0.5** | 0.269 | 0.117 | 0.98 | 0.681 | 0.751 |
|  | **0.6** | 0.172 | 0.149 | 0.975 | 0.555 | 0.847 |
|  | **0.7** | 0.096 | 0.195 | 0.97 | 0.404 | 0.919 |
| 38 weeks | **0.3** | 0.524 | 0.054 | 0.992 | 0.879 | 0.488 |
|  | **0.4** | 0.409 | 0.063 | 0.989 | 0.802 | 0.604 |
|  | **0.5** | 0.276 | 0.081 | 0.986 | 0.692 | 0.738 |
|  | **0.6** | 0.162 | 0.113 | 0.983 | 0.569 | 0.851 |
|  | **0.7** | 0.079 | 0.152 | 0.978 | 0.374 | 0.93 |
